# Participation during First Social Encounters in Schizophrenia

**DOI:** 10.1371/journal.pone.0077506

**Published:** 2014-01-20

**Authors:** Mary Lavelle, Patrick G. T. Healey, Rosemarie McCabe

**Affiliations:** 1 Department of Health Service and Population Research, Institute of Psychiatry, King’s College London, London, United Kingdom; 2 Interaction, Media and Communication Research Group, School of Electronic Engineering & Computer Science, Queen Mary, University of London, London, United Kingdom; 3 University of Exeter Medical School, Exeter, United Kingdom; Institute of Psychiatry at the Federal University of Rio de Janeiro, Brazil

## Abstract

**Background:**

Patients with a diagnosis of schizophrenia are socially excluded. The aim of this study was to investigate how patients participate in first encounters with unfamiliar healthy participants, who are unaware of their diagnosis.

**Methods:**

Patterns of participation were investigated during interactions involving three-people. Three conversation roles were analysed: (i) speaker, (ii) primary recipient- focus of the speaker’s attention and (iii) secondary recipient- unaddressed individual. Twenty patient interactions (1 patient, 2 healthy controls) and 20 control interactions (3 healthy participants) were recorded and motion captured in 3D. The participation of patients and their partners, in each conversation role, was compared with controls at the start, middle and end of the interaction. The relationship between patients’ participation, their symptoms and the rapport others experienced with them was also explored.

**Results:**

At the start of the interaction patients spoke less (ß* = *−.639, *p* = .02) and spent more time as secondary recipient (ß* = *.349, *p* = .02). Patients’ participation at the middle and end of the interaction did not differ from controls. Patients’ partners experienced poorer rapport with patients who spent more time as a primary recipient at the start of the interaction (Rho(11) = −.755, *p*<.01). Patients’ participation was not associated with symptoms.

**Conclusion:**

Despite their increased participation over time, patients’ initial participation appears to be associated with others’ experience of rapport with them. Thus, the opening moments of patients’ first encounters appear to be interpersonally significant. Further investigation of patient and others’ behaviour during these critical moments is warranted in order to understand, and possibly develop interventions to address, the difficulties schizophrenia patients experience here.

## Introduction

A prominent feature of schizophrenia is patients’ social exclusion. Patients have smaller social networks, less satisfactory interpersonal relationships and greater unemployment than healthy people or patients with other psychiatric disorders [1], [2]. A central and debilitating feature of schizophrenia, which may contribute to patients’ social exclusion, is patients’ difficulty interacting with others. These social deficits are poorly understood. Patients display poor performance on ‘off-line’ assessments of social cognition, which investigates the ability to discriminate facial expressions in pictures, attribute emotional states to the protagonists in short narratives and infer intentions in abstract problem solving contexts [3]. However, how patients’ social deficits manifest during their ‘on-line’ social interactions with others remains largely unexplored.

Early psychiatrists described feeling an intuitive ‘lack of rapport’ when interacting with a patient with schizophrenia, which was termed the ‘Praecox feeling’ [4]. According to Rümke (1941) the praecox feeling is based on patients’ nonverbal behaviour. This may extend beyond patients’ clinical interactions and influence their everyday social interactions. Indeed, a recent study revealed that patients’ nonverbal behaviour (e.g. hand and head movement) during first encounters with unfamiliar others, influenced others’ experience of rapport with the patient [5]. This was the case even though others were unaware of the patient’s diagnosis. Thus, alongside stigma due to others’ knowledge of the disorder, patients’ behaviour during social interactions may contribute to others’ experience of the interaction.

The aim of this study is to assess patients’ participation during first encounters with others. The study of three-person interactions provides an ideal interaction setting in that, unlike two-person interactions where individuals are obliged to take the role of the speaker or hearer, two hearer roles are possible: (i) a primary recipient: the individual who the speaker is addressing and directing their speech towards and (ii) a secondary recipient: a fully ratified participant who the speaker is not currently addressing [6], [7]. The speaker and primary recipient form the ‘active pair’, sharing a particular relationship with increased mutual gaze and coordinated movement, which the unaddressed, secondary recipient is not party to [7], [8].

Here we investigate how, during first encounters, patients participate in interactions with two others who are unaware of their diagnosis. In line with previous studies investigating multiparty interaction, the identity of primary and secondary recipients in the current study will be approximated from speakers’ head angle, using 3-D motion capture data [5], [7], [9]. The relationship between patients’ participation, their symptoms and the rapport their partners experience with them will also be explored.

## Methods

### Ethics Statement

All procedures were approved by the Charing Cross Research Ethics Committee (London, UK: 07/H0711/90) and all participants provided written informed consent.

### Study Design

The current study is a secondary analysis of data collected to investigate social interaction in schizophrenia [5]. The corpus under analysis involved three-way interactions in two conditions: A patient condition, involving one patient with a diagnosis of schizophrenia and two healthy participants (i.e. patients’ partners), and a control condition, involving three healthy participants (i.e. controls). This study analysed the amount of time participants spent as a speaker, primary and secondary recipients.

### Sample

Twenty patients with a diagnosis of schizophrenia (6 male, 14 female) and one hundred non-psychiatric healthy participants, forty in the patient condition (21 male, 19 female) and sixty in the control condition (34 male, 26 female), participated in the study. Recruitment and data collection occurred from July 2008 to Nov 2010. Patients were recruited at routine psychiatric outpatient clinics, on the basis of a clinical diagnosis of schizophrenia. Of all patients approached, 25% agreed to participate. Diagnosis was confirmed using the Structured Clinical Interview for DSM-IV symptoms [10]. Patients presenting with motor side effects from anti-psychotic medication (e.g. muscle stiffness and involuntary muscle spasms) were excluded from the study based on clinicians’ assessment. Patients’ antipsychotic medication was documented and converted into chlorpromazine equivalents (CPZE mg/day) according to the standard formula suggested by Woods [11].

Non-fluent English speakers were also excluded. Non-psychiatric healthy participants were recruited through advertising on local community websites. Of those who responded to the advertisement, 40% participated. Participants with a diagnosis of psychosis or affective disorders in themselves, or any first-degree relatives, and those who were not fluent English speakers were excluded. Interacting participants had not met prior to the study. Healthy participants were informed that the study was an investigation of social interaction, and were not aware that a psychiatric patient was present. All interactions were conducted outside of a psychiatric department, i.e. in a non-medical university department. After complete description of the study to the subjects, written informed consent was obtained.

### Assessments

Patients’ diagnosis of schizophrenia, paranoid subtype, was confirmed using the Structured Clinical Interview for Diagnostic symptoms (SCID-I) [10]. Patients’ positive, negative and general symptoms were assessed using the Positive And Negative Syndrome Scale for schizophrenia (PANSS) [12]. The Brixton Spatial Anticipation Test and the Hayling Sentence Completion Test assessed participants executive functioning [13]. Participants rated the level of rapport or connection they felt with each interacting partner on a 10 point scale, with a higher score indicating stronger rapport [14].

### 3D Motion-capture Equipment

The Augmented Human Interaction laboratory, where interactions were recorded, was fitted with an optical based Vicon motion-capture system, consisting of 12 infrared cameras and Vicon iQ software. Participants wore a top and cap with 27 reflective markers attached. The motion capture system detected the precise spatial and temporal coordinates of participants’ markers in 3D at each frame of the interaction, at a rate of 60 frames per second. This information was exported in numerical values and as a wire-frame representation of the interaction over time.

### Procedure

Each group of three participants were seated and asked to discuss a moral dilemma called ‘The Balloon Task’ [15]. During a one-to-one interview with a researcher, all participants’ socio-demographic and executive functioning was assessed. Patients’ clinical features and social functioning were also assessed. A detailed description of the procedure is described elsewhere [5].

#### Identification of speaker and hearers

**Figure 1:**
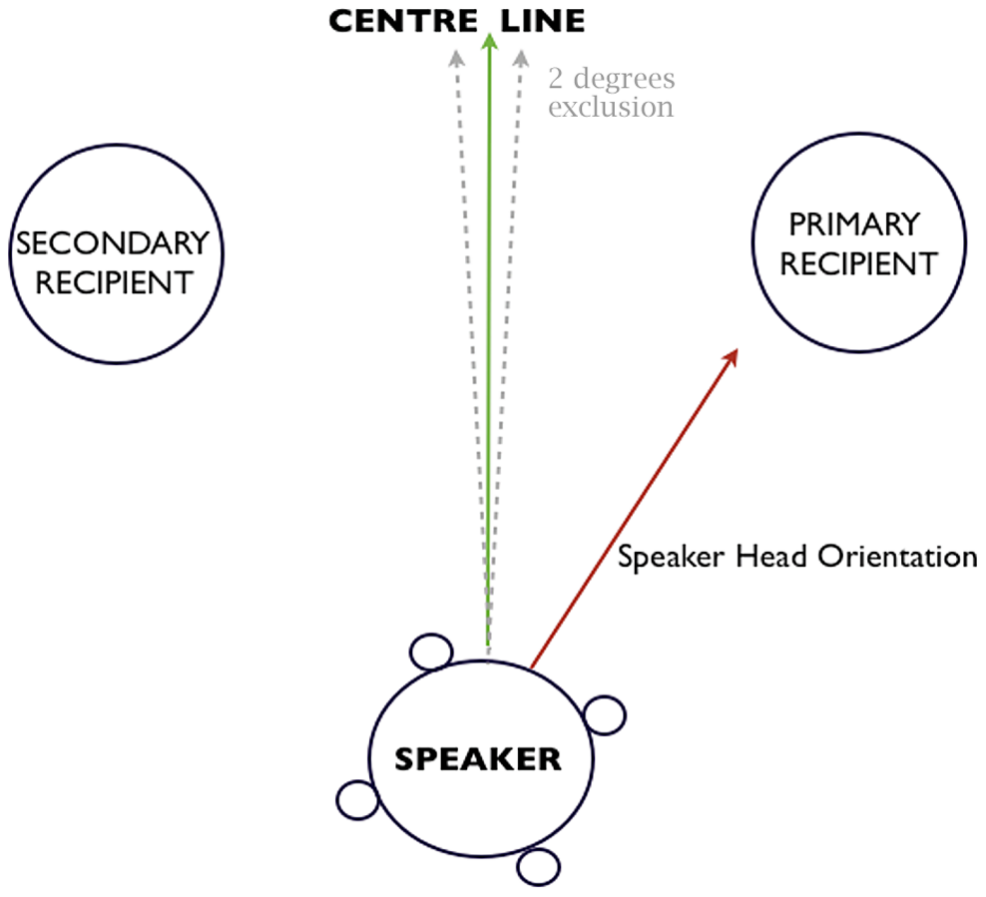
Indexing primary and secondary recipient from the head angle of the speaker. Speaker’s head angle is derived from the precise positions (3D) of the four reflective markers attached to their cap.

The identification of the speaker, primary and secondary recipients was assessed on a frame-by-frame basis. Speakers were hand coded from 2D videos using ELAN annotational software [16]. Primary recipients were identified as the person the speaker was oriented towards. Speakers’ head orientation was determined based on the precise spatial coordinates of the four optical markers attached to their caps (see figure 1) [7]. This procedure has been used in previous studies to index listener role in three-way interactions [7]. Frames with speaker head orientations falling less than two degrees from the centre line were excluded from the analysis (15% of the data set) to eliminate ambiguity.

### Statistical Analysis

Statistical analyses were conducted using SPSS Version 20 (© Copyright SPSS Inc. 2010). Independent samples t-tests were used to compare interaction duration (in seconds) between conditions, and age and executive functioning between patients and healthy participants. Chi 2-tests compared: the distribution of gender across conditions (i.e. patient condition (*n* = 60) and control condition (*n* = 60) and between patients (*n* = 20) and healthy participants (*n = *100).

#### Conversation role

In order to identify change in conversational roles over time, the percentage of time spent in each role was assessed at three time points during the interaction (i.e. the start, middle and end of the interaction). In order to adjust for the variations in total interaction duration between groups, each interaction was divided into ten sections based on the interaction duration (i.e. interaction duration (seconds)/10). Each section had a mean duration of approximately 30 seconds. The percentage of time participants spent in each conversation role was calculated at sections one (interaction start: approximately 0–30 seconds) five (interaction middle: approximately 120–150 seconds) and eight (interaction end: approximately 210–240 seconds). The final two sections (approx 1 minute) of the interactions were not analysed as observations revealed a trailing off during this final minute, so patterns of participation may be misrepresented in this data.

Mixed models analyses compared percentage of time spent in each conversation role between the three participant types (patient, patients’ partners and controls) at the start (section 1), middle (section 5) and end (section 8) of the interaction, clustering for the three-person group participants belonged to. Participants’ age, gender and executive functioning were accounted for in the model and reported if significant.

#### Patients’ conversation role, clinical features and rapport

Spearman-Rho nonparametric correlation coefficients (two-tailed) assessed the relationship between percentage of time patients spend in each conversation role (speaker, primary recipient and secondary recipient) and patients’ symptoms (PANSS), antipsychotic medication dose (CPZE), and the rapport others experience with them. Significant associations were further investigated using regression analyses.

## Results

Interactions lasted on average 5 minutes 24 seconds (SD = 1 minute 55 seconds). The duration of interactions in the patient condition (M = 318.08 sec, SD = 113.35) did not differ from the control condition (M = 335.29 sec, SD = 118.91) (t(38) = −4.62, *p = *.65).

The distribution of gender in the patient condition (*n* = 60: female = 53.33%) and control condition (*n* = 60: female = 43.33%) did not significantly differ (X^2^ (1) = 1.20, p = .27). The distribution of gender within patients (*n* = 20: female = 65%) and healthy participants (*n = *100: female = 45%) did not significantly differ (X^2^ (1) = 2.67, p = .10). Participants’ age and scores on assessments of executive functioning are displayed in table 1. Compared to healthy participants, patients were older (p<.01) and displayed poorer executive functioning (p<.01).

**Table 1:** Age and executive functioning by participant type.

	Schizophrenia patients (n = 20)		Healthy participants (n = 100)				
Variables	M	SD	M	SD	*t*	*df*	*p*
Age	41.50	8.64	31.10	9.60	−4.51	119	<.01
Spatial Executive functioning	3.07	0.51	5.10	0.22	3.65	119	<.01
Verbal Executive functioning	3.79	0.43	4.91	0.18	2.52	119	<.01

### Patients’ Clinical Features

Patients’ mean duration of illness was 15 years (SD = 10.26; range 2–46 years), with an average inpatient admissions duration of 19.95 weeks (SD = 18.45; range 0–60). Patients’ mean symptom scores were: positive 15.80 (SD = 6.76; range 7–37) negative 9.95 (SD = 3.36; range 7–19) and general symptoms 28.41 (SD = 10⋅42; range 16–59). Three patients were medication free at the time of the study and the remaining seventeen were taking typical (2 patients) or atypical (15 patients) antipsychotic medication. Of those taking antipsychotic medication, the average dose was 167.87 mg/day (SD = 109.29; range 50–400 mg/day), which fell within the low dose range (CPZE = 50–200 mg/day) [17].

### Conversation Role

**Figure 2:**
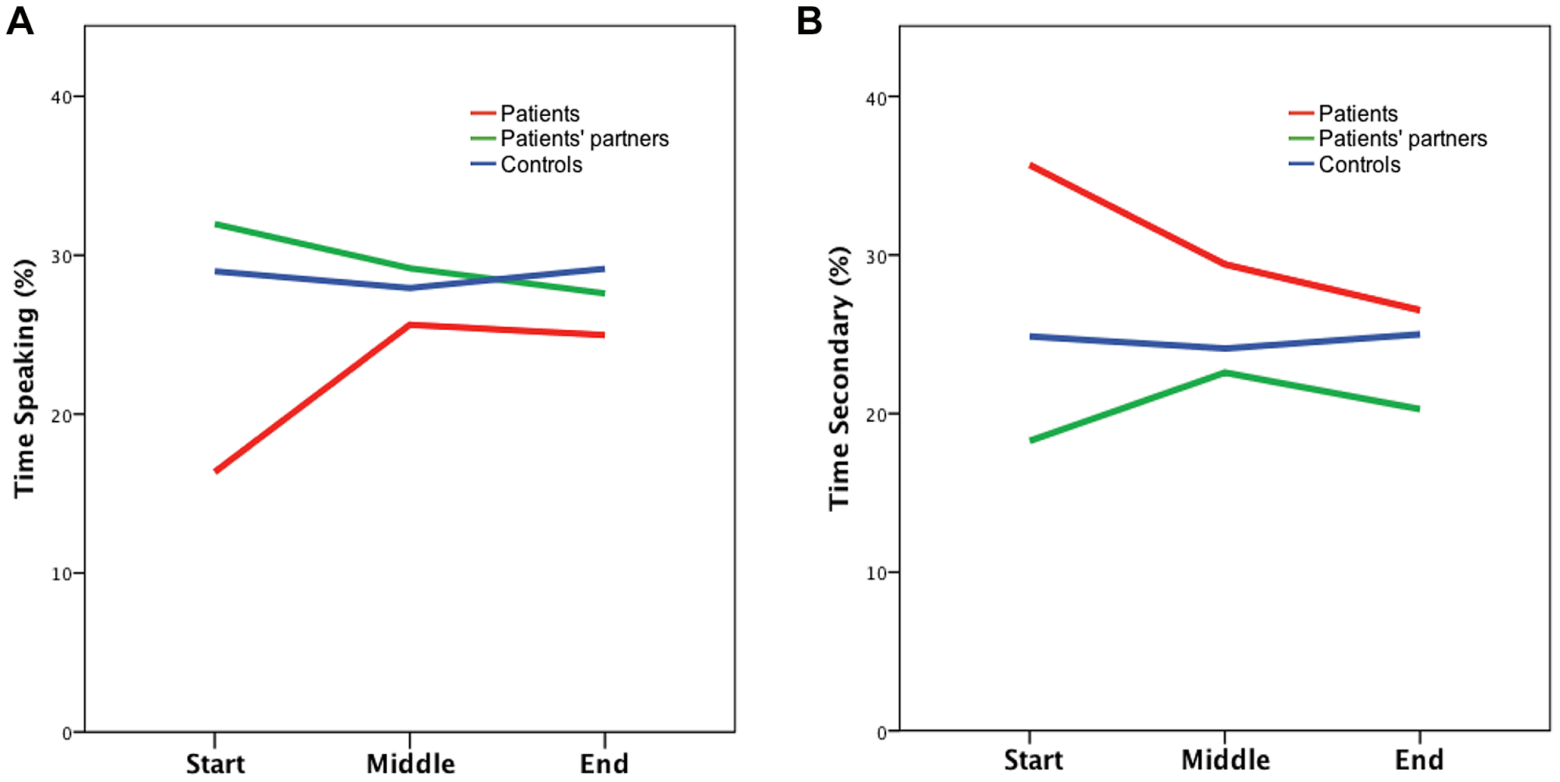
Mean percentage of time spent as speaker (A) and secondary recipient (B) at the start, middle and end of the interaction by participant type.

Mixed models comparisons of conversation role, by participant type, at each time interval are displayed in table 2. At the start of the interaction, compared to participants in the control group, patients spent less time speaking (ß* = *−.639, *p* = .02) and more time in the role of secondary recipient (ß* = *.349, *p* = .02), while patients’ partners spent less time as a secondary recipient (ß* = *−.357, *p* = .02). Compared to male participants, female participants spent more time in role of secondary recipient at the start of the interaction (*p* = .01), and less time as a primary recipient at the end of the interaction (*p* = .05). Patients or their partners did not differ from controls in the amount of time they spent in any conversation role at the middle or end of the interaction (*p*>.05). Figure 2 displays the mean percentage of time spent as speaker and secondary recipient at each time point, by participant type.

**Table 2:** Mixed models analyses investigating the percentage of time spent in each conversation role by participant type at the start, middle and end of the interaction.

						95% CI				
Models	*n*	M (%)	SE	ß	SE	Lower	Upper	Chi^2^	*df*	*p*
**Interaction start**										
**1. Speaking**										
Patients	20	17.35	3.81	−.639	.28	−1.186	−.093	5.26	1	.02
Patients’ partners	40	33.22	1.73	.141	.10	−.072	.355	1.68	1	.19
Controls	60	29.97	0.94							
**2. Primary**										
Patients	20	21.96	3.70	−.197	.23	−.657	.264	0.70	1	.40
Patients’ partners	40	26.27	2.37	−.111	.14	−.377	.155	0.67	1	.41
Controls	60	26.64	0.74							
**3. Secondary**										
Patients	20	36.38	5.36	.349	.15	.054	.645	5.36	1	.02
Patients’ partners	40	17.96	2.67	−.357	.15	−.659	−.055	5.36	1	.02
Controls	60	25.66	0.81							
Gender: Male	62	20.64	2.20	−.430	.17	−.754	−.107	6.79	1	.01
Gender: Female	58	31.73	2.45							
**Interaction middle**										
**4. Speaking**										
Patients	20	26.62	5.58	−.018	.26	−.533	.498	0.01	1	.94
Patients’ partners	40	29.98	2.68	−.069	.12	−.165	.303	0.34	1	.56
Controls	60	29.13	2.62							
**5. Primary**										
Patients	20	20.75	3.85	−.305	.21	−.716	.106	2.11	1	.15
Patients’ partners	40	27.38	3.05	.050	.16	−.261	.361	0.10	1	.75
Controls	60	25.68	2.65							
**6. Secondary**										
Patients	20	30.41	4.06	.189	.27	−.331	.709	0.51	1	.48
Patients’ partners	40	22.89	3.10	−.002	.14	−.282	.277	0.01	1	.99
Controls	60	25.49	2.84							
**Interaction end**										
**7. Speaking**										
Patients	20	25.97	5.22	−.148	.28	−.700	.404	0.28	1	.60
Patients’ partners	40	28.13	2.62	−.040	.12	−.276	.196	0.11	1	.74
Controls	60	30.38	1.11							
**8. Primary**										
Patients	20	19.97	4.77	−.239	.29	−.813	.336	0.66	1	.42
Patients’ partners	40	23.98	3.15	−.244	.20	−.645	.157	1.43	1	.23
Controls	60	26.96	0.99							
Gender: Male	62	26.90	2.41	.278	.14	.006	.551	4.02	1	.05
Gender: Female	58	20.36	1.85							
**9. Secondary**										
Patients	20	27.52	5.12	−.060	.30	−.639	.520	0.04	1	.84
Patients’ partners	40	20.55	2.92	−.130	.17	−.469	.209	0.56	1	.45
Controls	60	26.23	0.81							

**Key:** Patients’ partners – Healthy participants in the patient condition; Controls – Healthy participants in the control condition.

**Model 1.** Goodness of fit QIC = 68.35; **Model 2.** Goodness of fit QIC = 74.67; **Model 3.** Goodness of fit QIC = 119.0.

**Model 4.** Goodness of fit QIC = 76.00; **Model 5.** Goodness of fit QIC = 80.57; **Model 6.** Goodness of fit QIC = 100.19. **Model 7.** Goodness of fit QIC = 80.91; **Model 8.** Goodness of fit QIC = 94.46; **Model 9.** Goodness of fit QIC = 84.10.

#### Conversation role and clinical features

Spearman-Rho correlations found no significant association between the time patients spent in each conversation role (start middle or end) and their symptoms (positive, negative or general) (*p*>.05) or antipsychotic medication dose (*p*>.05).

#### Conversation role and rapport

Spearman-Rho correlation revealed that patients’ partners experienced poorer rapport with patients who, at the start of the interaction, spent less time in the role of secondary recipient (Rho(11) = .705, *p* = .02) and more time in the role of primary recipient (Rho(11) = −.755, *p*<.01).

## Discussion

The findings revealed that, at the start of a first encounter, patients spoke less and spent more time as a secondary recipient. However, as the interaction progressed patients’ participation in the conversation did not differ from controls. Patients’ partners experienced poorer rapport with patients who, at the start of the interaction, spent more time as the focus of the speaker’s attention, in the role of primary recipient. Patients’ symptoms were not associated with their participation at any time interval.

In the opening moments of the interaction, patients spent less time speaking and more time in the role of secondary recipient. This suggests that patients are taking a more peripheral role at the start of the interaction, speaking, and being spoken to, less. In line with these findings, previous studies identified that schizophrenia patients show increased ‘flight’ behaviour, signaling the avoidance of social stimuli, during the first two minutes of a clinical consultation [18]. Thus, it appears that schizophrenia patients display signs of social withdrawal at the start of an interaction. Patients may find participating in a face-to-face interaction demanding due to social anxiety. This may have implications for devising interventions to address patients’ initial difficulties settling into a conversation with others, both in and outside of clinical contexts.

Differences between patients and controls were identified after adjusting for gender. However, investigation across all participants revealed differences in participation between gender. Specifically, compared to males, females were more likely to be secondary recipients at the start of the interaction and less likely to be primary recipients at the end. The size of the patient sample in the current study was insufficient to investigate any interaction effects for patient and gender. However, this should be considered in future studies.

Patients’ participation at the middle or end of the interaction did not differ from healthy participants in the control condition. This suggests that over the course of an interaction, patients become more actively involved as a speaker and spend less time in the role of secondary recipient. Micro-analyses of patients’ three-way interactions has found that when patients are reluctant to speak, others actively attempt to involve them in interaction: initially using subtle nonverbal cues and, if unsuccessful, verbally requesting their input [19]. Perhaps patients’ increased involvement over the course of the interaction is a result of both others actively attempting to engage the patient and patients becoming more comfortable in the interaction. Patients’ increased participation over time suggests that, rather than patients being excluded from the speaker-primary recipient pair over the course of a first meeting, patients initially appear to participate less but become more involved as the interaction progresses.

Others’ experience of rapport with the patient was associated with patients’ participation at the start of the interaction (approximately the first 30 seconds). Specifically, others experienced poorer rapport with patients who spent more time in the role of primary recipient and less time as secondary recipient. This suggests that patients’ partners are detecting something in the patient’s behaviour at the start of the interaction that is making them more likely to be the focus of their attention (i.e. primary recipient). In a previous analysis of this corpus, patients’ increased hand movement during interaction was associated with others’ poorer experience of rapport with them [5]. Thus, despite the fact that patients’ participation improves over time, it appears that the opening moments of patients’ first encounters are interpersonally important. Future research should further investigate other aspects of patients’ behaviour (e.g., what they are saying, their nonverbal behaviour) and their partners’ responses to them at this critical phase in the interaction. Moreover, understanding how patterns of participation develop over longer and repeat encounters would also be of relevance in understanding social integration of patients.

The current study found no association between patients’ participation in conversation and their symptoms. It might have been expected that patients with more negative symptoms would spend less time speaking. However, the variance on patients’ symptoms in the current sample was low, which may have impeded the ability to detect an association if one existed.

### Strengths and Limitations

This study benefited from the three-person experimental design, enabling the investigation of patients’ participation in contexts more typical of everyday social interaction. Furthermore, by ensuring all interacting partners were unfamiliar to each other and unaware of patients’ diagnoses, the possible confounding variables of familiarity and stigma, due to prior knowledge of the disorder, were removed. Although the interactions were unscripted conversations, they were recorded in a motion capture lab, which is a somewhat unnatural environment. Future studies could employ new motion capture techniques, such as the Kinect system, to obtain similar data from interactions outside a lab setting [20]. Examining patients’ interactions in more naturalistic environments may allow for recruitment of patients who are more symptomatic addressing the limited symptom variance seen in the current study. As the head angle of primary or secondary recipients was not investigated we cannot say whether the primary recipient is returning the gaze of the speaker. Although this was not necessary to answer the questions of the current study, future studies could investigate patients’ reciprocation of speakers’ gaze in the role of primary recipient.

### Conclusion

The aim of this study was to investigate schizophrenia patients’ participation during first encounters with unfamiliar others who were unaware of their diagnosis. The findings indicate that although patients take a more peripheral role at the start of the interaction, they participate more as the interaction progresses. Despite this, it is the first 30 seconds of the encounter that influences others’ experience of rapport with them. This highlights the opening moments of patients’ conversations as interpersonally significant. Future research should identify the precise behaviours displayed, by patients and others, during this critical period in order to develop our understanding of patients’ social deficits and their impact on interpersonal relationships.
